# A Rare Case of Bilateral Heel Sore Flap Cover

**Published:** 2018-05

**Authors:** Chetan Satish

**Affiliations:** RT Nagar, Bangalore, India

**Keywords:** Bilateral, Heel, Sore, Flap

**DEAR EDITOR**

Different flaps are available for heel reconstruction, including medial plantar flap involving the medial plantar nerve and its cutaneous branches, which may result to postoperative hyperesthesia and dual sensation.^1^ Full-thickness defects to the plantar surface of the foot are still a challenge in plastic surgery. Skin grafts and many flap procedures were used to resurface this site, but not all achieve a return to normal foot function. For the heel plantar surface, medial plantar flap has been used, however, this method leaves a donor site, which requires skin grafting. The modification of the medial plantar flap into a V-Y configuration has been reported allowing the direct closure of the donor site.^2^ Here, a rare case of bilateral heel sores following long standing bed-ridden state has been reported that was successfully treated with bilateral reverse sural artery flaps. Although reverse sural artery flap is commonly used for heel cover, no case of bilateral heel pad cover at same sitting has been reported.

A 20-year old male patient with bilateral heel sores was referred for wound cover. The patient had been admitted with history of poly-trauma about 5 months ago. He had multiple long bone fractures with head injury and had undergone multiple surgeries in his protracted hospital stay. He had also developed Acute respiratory distress syndrome and ultimately recovered from his other conditions. He had not been mobilized in view of his lung condition and long bone fractures. The orthopedic team had opined another 1 month of immobilzation for his long bone fractures when he was referred for flap cover. 

He had bilateral heel pressure sores with tendo-achilles exposed on both legs. He underwent bilateral islanded reverse sural flap cover after pre-operative Doppler study. The procedure took about 3 hours and was done in prone position. His pre-operative photographs are shown in Figure 1. He was nursed in prone position post-operatively and the wounds healed in about 3 weeks. Post-operative photographs are shown in Figure 2. At present the patient is mobilized and is able to walk normally.

**Fig. 1:**
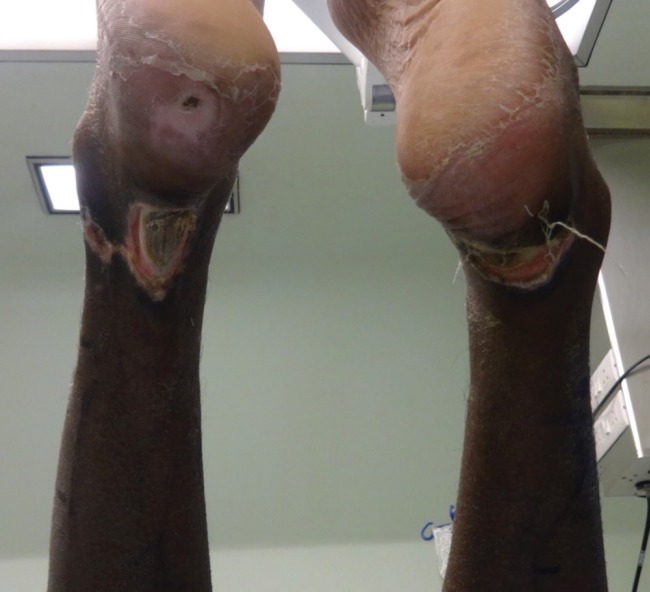
The pre-operative photograph

**Fig. 2:**
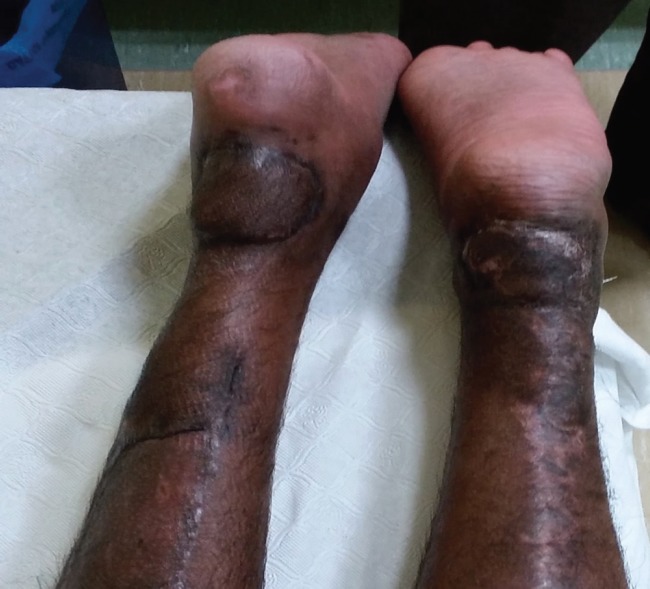
Post-operative photograph

Heel pressure sores are relatively common in bedridden patients. 

They need to be prevented by proper precautions including air beds, frequent change of positions and early mobilization. However, in case these measures have failed and patient develops heel sores flap cover is advised at earliest. This is to facilitate early mobilization and to salvage the tendo-achilles tendon. Heel sores have been treated with medial plantar artery flap^3^ and by the reverse sural artery flap.^4^ Bilateral heel pressure sores can be treated simultaneously in single sitting with flap cover which prevents time delay and need for multiple surgeries. Islanded reverse sural flap is a very reliable flap for heel sores and the reliability can be improved by pre-operative Doppler markings of perforators.^3^^,^^4^ Heel pressure sores need to be identified at earliest and treated with flap cover. Islanded reverse sural flap is an ideal flap for heel sores with tendo-achilles tendon exposed. Successful flap coverage of bilateral heel sores in a single sitting has not been reported and should be considered as shown in this case.

## CONFLICT OF INTEREST

The authors declare no conflict of interest.
